# Ars2 promotes cell proliferation and tumorigenicity in glioblastoma through regulating miR-6798-3p

**DOI:** 10.1038/s41598-018-33905-x

**Published:** 2018-10-22

**Authors:** Yibiao Chen, Xiaoye Hu, Yunong Li, Hongwei Zhang, Ruoqiu Fu, Yanxia Liu, Jinjiao Hu, Qin Deng, Qingsong Luo, Dunke Zhang, Ning Gao, Hongjuan Cui

**Affiliations:** 1 State Key Laboratory of Silkworm Genome Biology, Southwest University, Chongqing, China; 2 College of Pharmacy, Third Military Medical University, Chongqing, China

## Abstract

Arsenic resistance protein 2 (Ars2) is a component of the nuclear RNA cap-binding complex (CBC) that is important for some microRNA biogenesis and it is critical for cell proliferation and tumorigenicity. However, mechanism of Ars2-regulated cellular proliferation and tumorigenicity in glioblastoma has not been fully understood. Western blotting was used to detect the expressions of Ars2, p53, p21, and cleavage/activation of caspases-3 (C-Caspase 3). Microarray and Quantitative Real-time PCR (qRT-PCR) were performed to identify the Ars2-regulated microRNAs. Apoptosis assessed by flow cytometry analysis was used to evaluate the role of Ars2 in cells proliferation. The lentivirus-mediated gene knockdown approach was conducted to determine the function of Ars2. The orthotopic glioblastoma xenograft was used to demonstrate the role of Ars2 in glioblastoma growth *in vivo*. The high expression of Ars2 was observed in several glioblastoma cell lines and was significantly associated with poorer overall survival. Importantly, the overexpression of Ars2 promoted cell proliferation and colony formation in glioblastoma cells, whereas the depletion of Ars2 inhibited cell proliferation, colony formation, and tumor growth. Mechanistic study revealed that knockdown of Ars2 reduced the expression levels of miR-6798-3p, which was responsible for the up-regulation of p53 and p21, leading to apoptosis. Furthermore, the knockdown of Ars2 suppressed tumor growth in orthotopic glioblastoma xenograft model and significantly prolonged the survival time of the tumor-bearing mice. These findings identify a critical role for Ars2 in regulation of proliferation and tumorigenicity in glioblastoma and suggest that Ars2 could be a critical therapeutic target for glioblastoma intervention.

## Introduction

Malignant gliomas are the most common and deadly brain tumors^1^, associated with high rates of morbidity and mortality^2^. Glioblastoma is one of the most common and primary of malignant gliomas, with poor prognosis and few therapeutic advances in the last decade. The life expectancy of patients with glioblastoma using the current standard of care is about 14 months after diagnosis despite aggressive chemotherapies, radiation, and surgery^3–8^. Glioblastomas are characterized by genetic alterations large and small, affecting genes that control cell proliferation, angiogenesis, apoptosis and invasion^3,9–15^. However, the molecular mechanism how genetic alterations control cell proliferation and apoptosis are poorly understood. Therefore, the comprehensive elucidation of genetic alterations in glioblastomas could provide novel targets that might be used for therapeutic, diagnostic, or prognostic purposes of glioblastoma.

Arsenic resistance protein 2 (Ars2) is a nuclear protein encoded by the human SRRT gene and was originally cloned in a screen for cDNAs which could confer sodium arsenite resistance in a hamster cell line^16^. Ars2 is identified as a new factor in the RNA silencing machinery that functions in cell proliferation in mammals and antiviral defense in flies^16–20^. In mammals, a truncated version of Ars2 protein has been implicated in arsenic resistance, but the full-length of this protein can regulates cell proliferation through a possible effect on RNA metabolism^16,21,22^. Ars2 is also a component of the CBC that is important for some microRNAs (miRNA-21, miRNA-155, and let-7) biogenesis and critical for cellular proliferation^16^. Ars2 interacts directly with the assembled CBP20/80 cap complex to form a tertiary complex termed CBC^16,23^. It has been reported that Ars2 interacts with the nuclear cap-binding complex and it is a components of the nuclear pri-miRNA processing complex. Knockdown of Ars2 can be sufficient to reduce the pri-miRNA processing and miRNA expression levels of a number of miRNAs, miR-155, miR-21, and let-7 included, implicated in transformation^16,24^.

Recently, It also has been reported that Ars2 is overexpressed in human hepato cellular carcinoma (HCC) and cholangiocarcinoma and therefore considered Ars2 as a prognostic, diagnostic marker, even a potential target for therapeutic intervention^25,26^. However, little is known about the mechanism of Ars2’s role in these processes in human glioblastoma. In this study, we have determined the importance of Ars2 in human glioblastoma cells. Overexpression of Ars2 promoted cell proliferation and tumorigenicity, whereas depletion of Ars2 inhibited cell proliferation and tumorigenicity in glioblastoma. Mechanistical studies revealed that depletion of Ars2 is sufficient to reduce the levels of miRNA including miR-6798-3p, leading to up-regulation of p53 and p21, resulting in induction of apoptosis, and culminating in inhibition of tumorigenicity. These findings identify a critical role for Ars2 in regulation of proliferation and tumorigenicity in glioblastoma and suggest that Ars2 could be critical therapeutic target for glioblastoma intervention.

## Results

### Overexpression of Ars2 Is Prognostic of Poor Survival in Glioblastoma Patients

**Figure 1:**
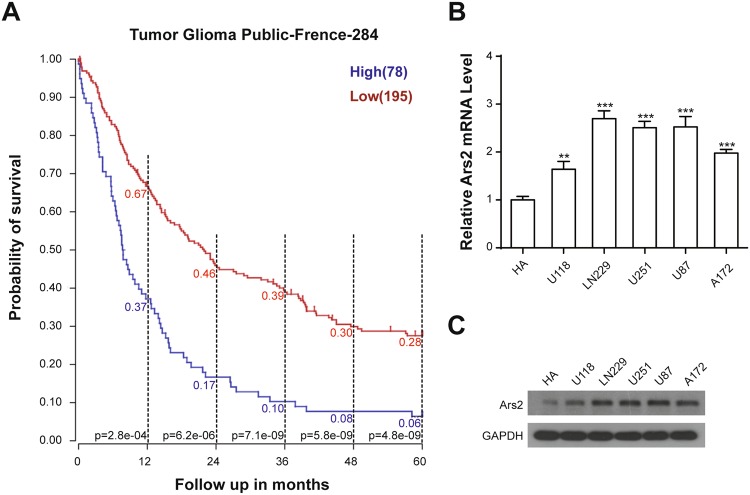
High expression of Ars2 in glioma patients and in glioblastoma cell lines is prognostic of worse survival in glioma patients. (**A**) Kaplan-Meier analysis of progression-free survival for the French database with the log-rank test *P* value indicated. Cutoff values for separating high and low expressing groups were determined by the online R2 database algorithm. (**B**) Five glioblastoma cell lines (U87, LN229, A172, U118, and U251) and normal human astrocytes (HA) were harvested and subjected to qRT-PCR analysis to detect Ars2 expression. Data were represented as the mean ± SD for three separate experiments, ^**^P < 0.01; ^***^P < 0.001. (**C**) The expression of Ars2 in five glioblastoma cell lines and HA was analyzed by Western blot analysis.

To determine the possibility of Ars2 as a prognostic marker for the poor survival glioblastoma patients, Kaplan-Meier analysis was employed to analyze the progression-free survival for the Frence database. As shown in Fig. 1A, the 3-year survival rates for patients with high Ars2 mRNA expression (78 cases) and low Ars2 mRNA expression (195 cases) were 10% and 39% (*p* < 0.001), respectively. The 5-year survival rates for patients with high Ars2 mRNA expression and low Ars2 mRNA expression were 6% and 28% (*p* < 0.001), respectively. These results suggest that high Ars2 expression was associated with poor prognosis, whereas low Ars2 expression was associated with better outcome. These results highlight the clinical importance of Ars2 in determining the prognosis for glioblastoma patients, indicating Ars2 as a new target for glioblastoma therapy. To determine the specific expression of Ars2 in human glioblastoma cell lines, quantitative real-time PCR (qRT-PCR) analysis was employed. As shown in Fig. 1B, Ars2 was highly expressed in 5 glioblastoma cell lines including U87, LN229, A172, U118, and U251 compared with normal human astrocytes (HA). Western blot analysis also confirmed the expression of Ars2 in these glioblastoma cell lines compared with HA (Fig. 1C). Together, these results indicate that the high expression of Ars2 in glioblastoma cells is prognostic of poor survival in glioblastoma patients.

### Overexpression of Ars2 Promotes Cell Proliferation and Colony Formation in Glioblastoma Cells

**Figure 2:**
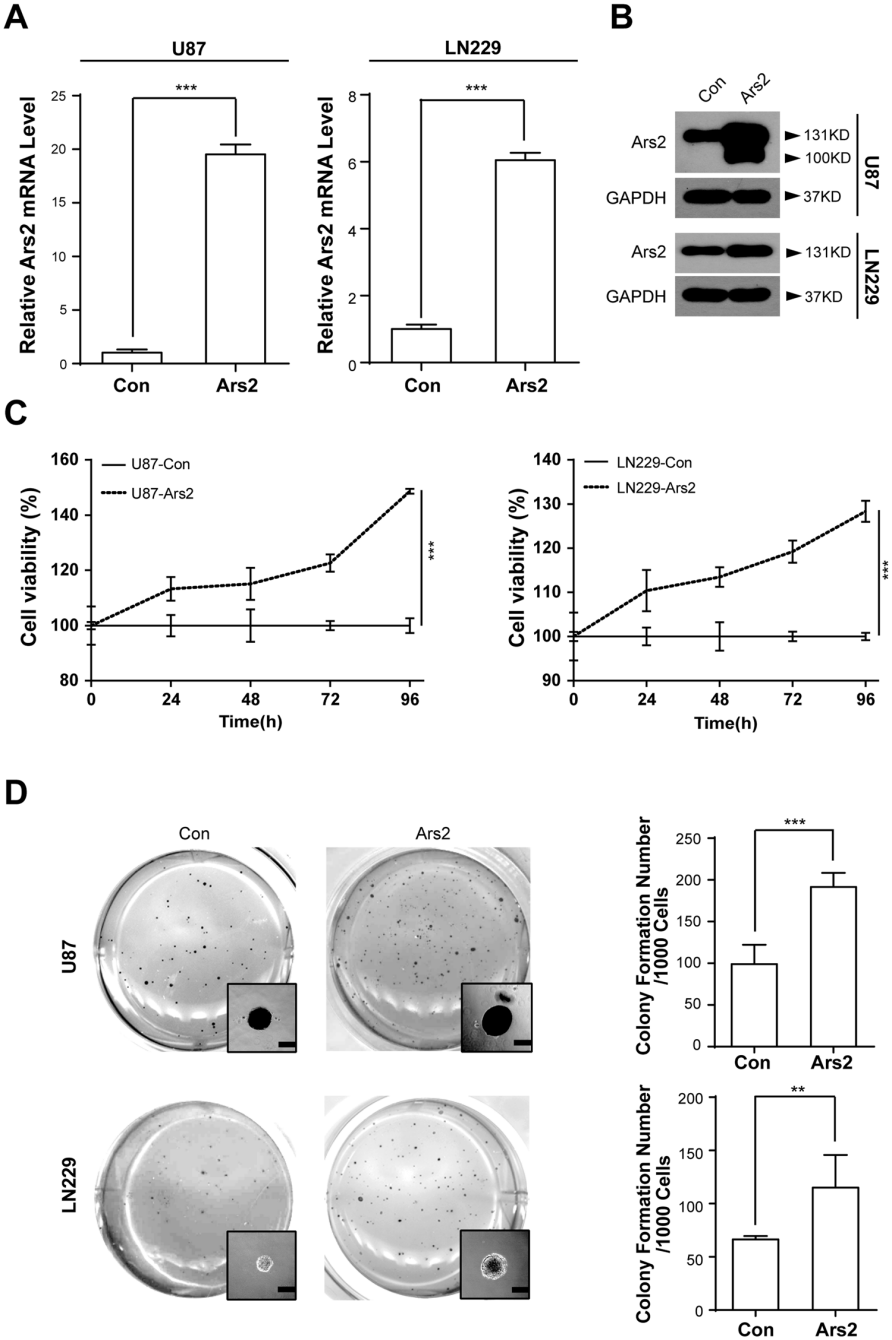
Ars2 promoted cell proliferation and colony formation in glioblastoma cells. U87 and LN229 cells were transfected with vector control and Ars2. (**A**) Total RNA was isolated and Ars2 mRNA was quantified using qRT-PCR analysis. (**B**) Western blot assay was used to detect the expression of Ars2 in U87-Con, U87-Ars2, LN229-Con, and LN229-Ars2 cells. (**C**) U87 cells and LN229 cells were seeded into a 96-well plate (1000 cells/well), and cell proliferation was determined by using MTT assay. (**D**) Colony formation in U87 cells and LN229 cells was detected by soft agar assay. All data were represented as the mean ± SD for three separate experiments, ^**^P < 0.01, ^***^P < 0.001.

To explore the functional role of Ars2 in cell proliferation and colony formation in glioblastoma cells, the stable Ars2-overexpression cells (U87-Ars2 and LN229-Ars2) were established by lentivirus-mediated infection of both U87 and LN229 cells with Ars2. qRT-PCR analysis showed that the mRNA levels of Ars2 in both Ars2-overexpressing cells (U87-Ars2 and LN229-Ars2) were 5 times higher than that in vector control cells (Fig. 2A). Western blot analysis also showed that the protein levels of Ars2 in Ars2-overexpressing cells were 2 times higher than that in vector control cells (Fig. 2B). We next performed MTT assay to investigate the effects of Ars2 overexpression on cell proliferation in both U87 and LN229 glioblastoma cells. As shown in Fig. 2C, Ars2 overexpression markedly promoted cell proliferation in both U87 and LN229 cells compared to that in vector control cells. To further uncover the effects of Ars2-overexpression on colony formation in both U87 and LN229 glioblastoma cells *in vitro*, soft agar assay was employed. As shown in Fig. 2D, the average size of colonies in Ars2 overexpressing cells was bigger than that in vector control cells. Furthermore, the number of colonies in Ars2 overexpressing cells was larger than that in vector control cells. Taken together, these results suggest that Ars2 plays a critical role in regulating cell proliferation and tumorigenesis in glioblastoma cells.

### Ars2 Depletion Suppresses Cell Proliferation and Colony Formationin in Glioblastoma Cells

**Figure 3:**
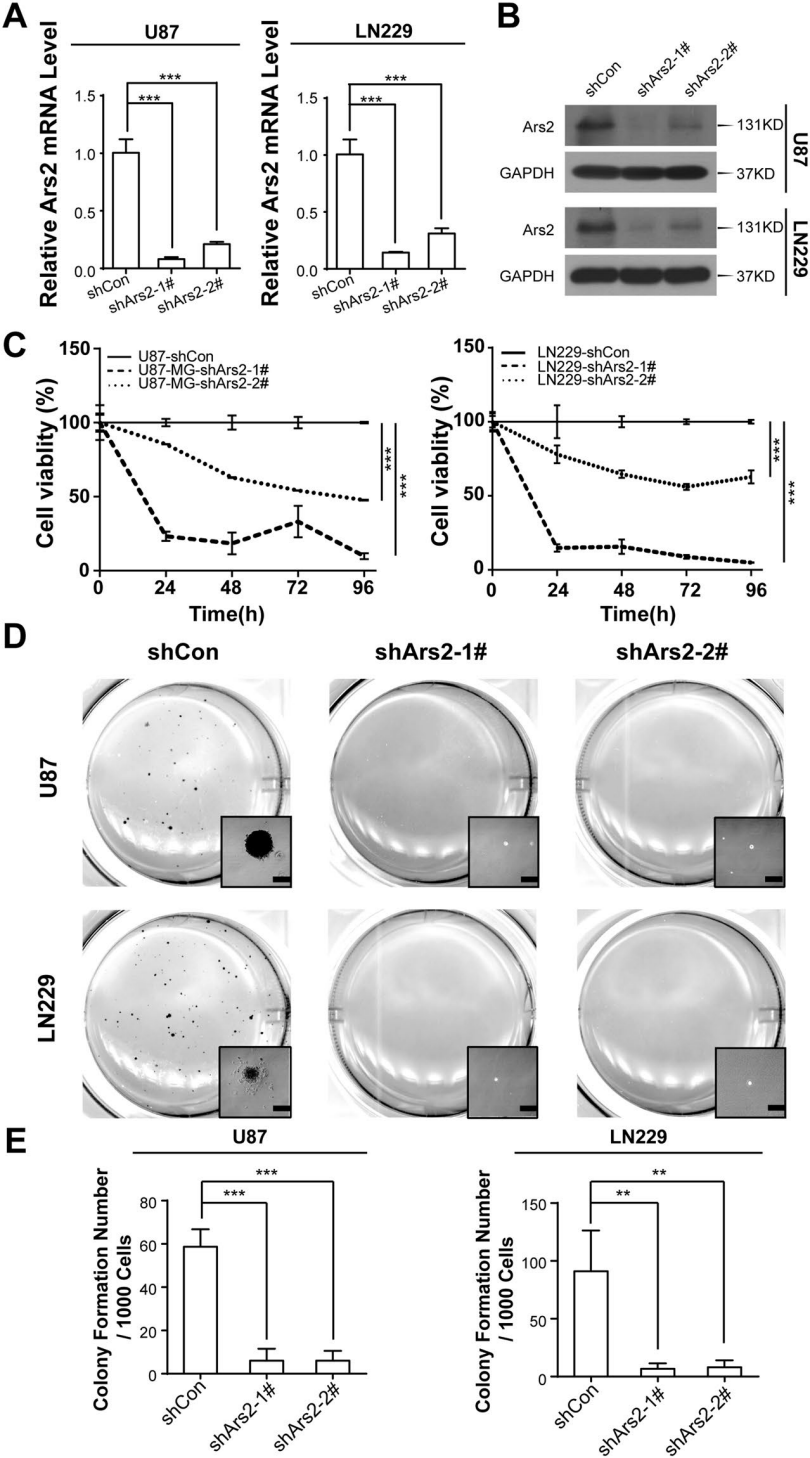
Depletion of Ars2 inhibited cell proliferation and colony formation in glioblastoma cells. U87 and LN229 cells were transfected with vector control siRNA (shCon) and Ars2 siRNA (shArs2-1#, and shArs2-2#). (**A**) Total RNA was isolated and Ars2 mRNA was quantified using qRT-PCR analysis. (**B**) Western blot assay was used to detect the expression of Ars2. (**C**) U87 and LN229 cells transfected with shCon and shArs2 were seeded into a 96-well plate (1000 cells/well), and cell proliferation was determined using MTT assay. (**D**,**E**) Colony formation was detected by soft agar assay. All data were represented as the mean ± SD for three separate experiments, ^**^P < 0.01, ^***^P < 0.001.

To further evaluate the functional role of Ars2 in cell proliferation and colony formation in glioblastoma cells, we knocked down Ars2 in U87 and LN229 cells by infecting with lentivirus-expressing shRNA targeting vector Ars2. Depletion of endogenous Ars2 was confirmed by qRT-PCR and Western blot analyses. As shown in Fig. 3A,B, the mRNA and protein levels of Ars2 were decreased in both U87 and LN229 cells infected with Ars2-shRNA compared with those in control shRNA cells. MTT assay showed that depletion of Ars2 with shRNA in both U87 and LN229 cells significantly suppressed cell proliferation compared with control shRNA cells (Fig. 3C). Furthermore, decreased numbers of colonies were observed in Ars2 shRNA cells compared to that in control shRNA cells (Fig. 3D,E). Taken together, these findings further confirm the important functional role of Ars2 in regulating cell proliferation and tumorigenesis in glioblastoma cells.

### Ars2 Depletion Induces Apoptosis in Glioblastoma Cells Through p53/p21 Dependent Pathway

**Figure 4:**
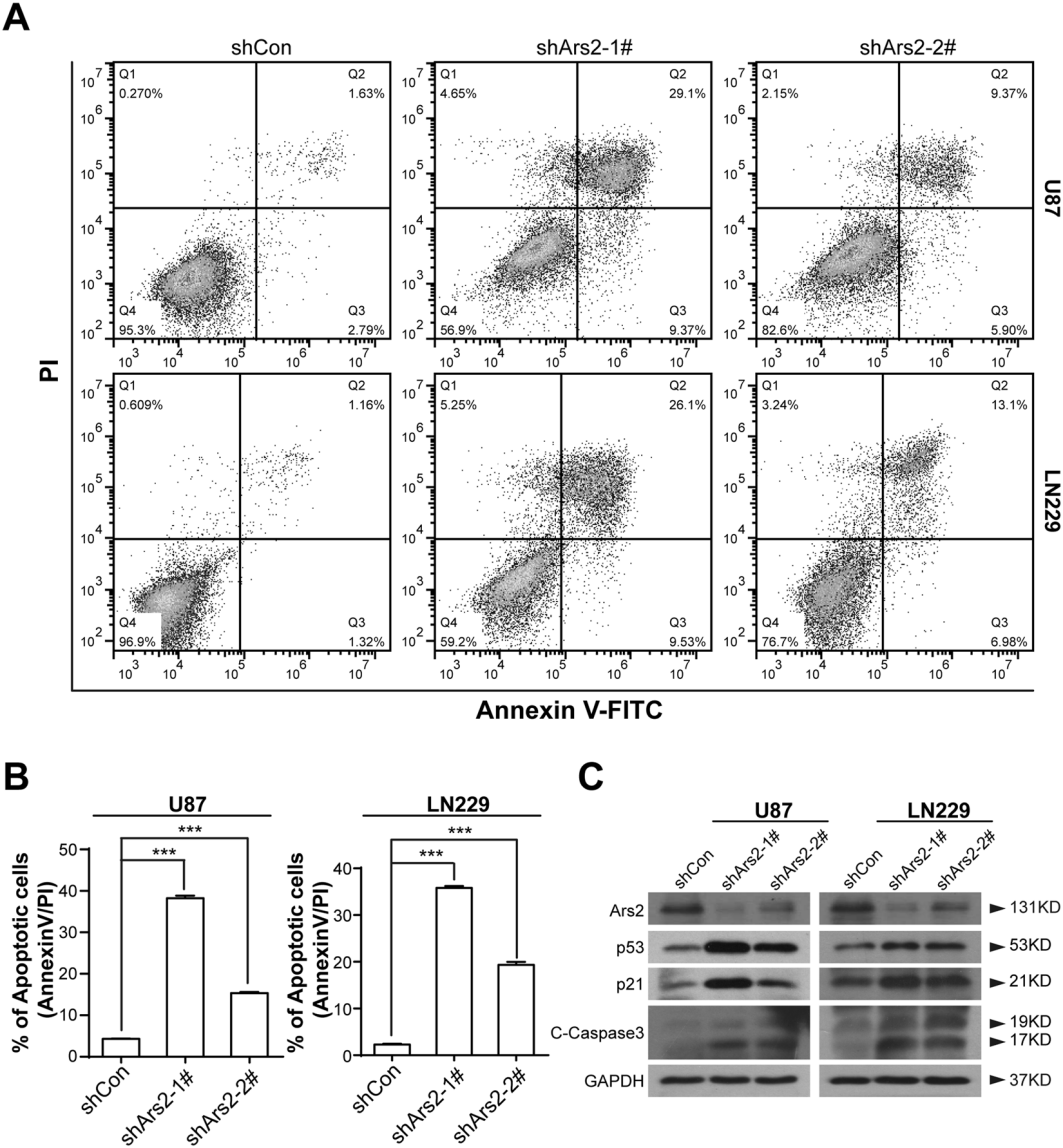
Depletion of Ars2 induced apoptosis in glioblastoma cells. U87 and LN229 cells were transfected with vector control siRNA (shCon) and Ars2 siRNA (shArs2-1#, and shArs2-2#). (**A**) The percentage of apoptotic cells was determined by flow cytometry using Annexin V/PI staining. (**B**) Statistical analysis of the cellular apoptosis levels. Data were represented as the mean ± SD for three separate experiments, ^***^P < 0.001. (**C**) Total cellular extracts were prepared and subjected to Western blot analysis using antibodies against p53, p21, and C-Caspase 3. GAPDH levels were shown as loading control.

To help understand how Ars2 functions in cell proliferation in detail, we investigated the effects of Ars2 depletion on apoptosis and cell cycle progression in U87 and LN229 glioblastoma cells by using flow cytometry. As shown in Fig. 4A,B, depletion of Ars2 with shRNA resulted in significantly increases in apoptosis in either U87 or LN229 cells. However, depletion of Ars2 did not affect cell cycle distribution in either U87 or LN229 cells (data not shown). Consistent with these findings, depletion of Ars2 with shRNA resulted in cleavage/activation of caspases-3 (Fig. 4C). In addition, cotreatment of cells with the caspase inhibitor z-VAD-fmk essentially abrogated apoptosis mediated by Ars2 depletion (Fig. S1A–C). To further confirm that the effect of Ars2 depletion on cell apoptosis is not an off-target, we next overexpressed Ars2 in Ars2-knocked down cells. As shown in Fig. S2A–C, overexpression of Ars2 essentially abrogated apoptosis mediated by Ars2 depletion.

Since p53 is a key tumor suppressor and plays an essential role in the regulation of cell apoptosis in glioblastoma cells^27,28^, we next investigated the effects of Ars2 depletion on the expression of p53 and its downstream target p21 by using Western blot analysis. We found that depletion of Ars2 with shRNA resulted in marked increase in levels of p53 and p21. Furthermore, overexpression of Ars2 markedly attenuated upregulation of p53 and p21 in Ars2 depletion cells (Fig. S2D). To further clarify whether p53/p21 pathway is involved in apoptosis mediated by Ars2 depletion, siRNAs with p53 and p21 were employed. As shown in Fig. S3A–C, knockdown of p53 or p21 markedly attenuated activation of caspase 3 and apoptosis mediated by Ars2 depletion. These results suggest that Ars2 depletion induces apoptosis in glioblastoma cells through p53/p21 dependent pathway.

### Ars2 Depletion Suppresses the Expression of miR-6798-3p in Glioblastoma Cells

**Figure 5:**
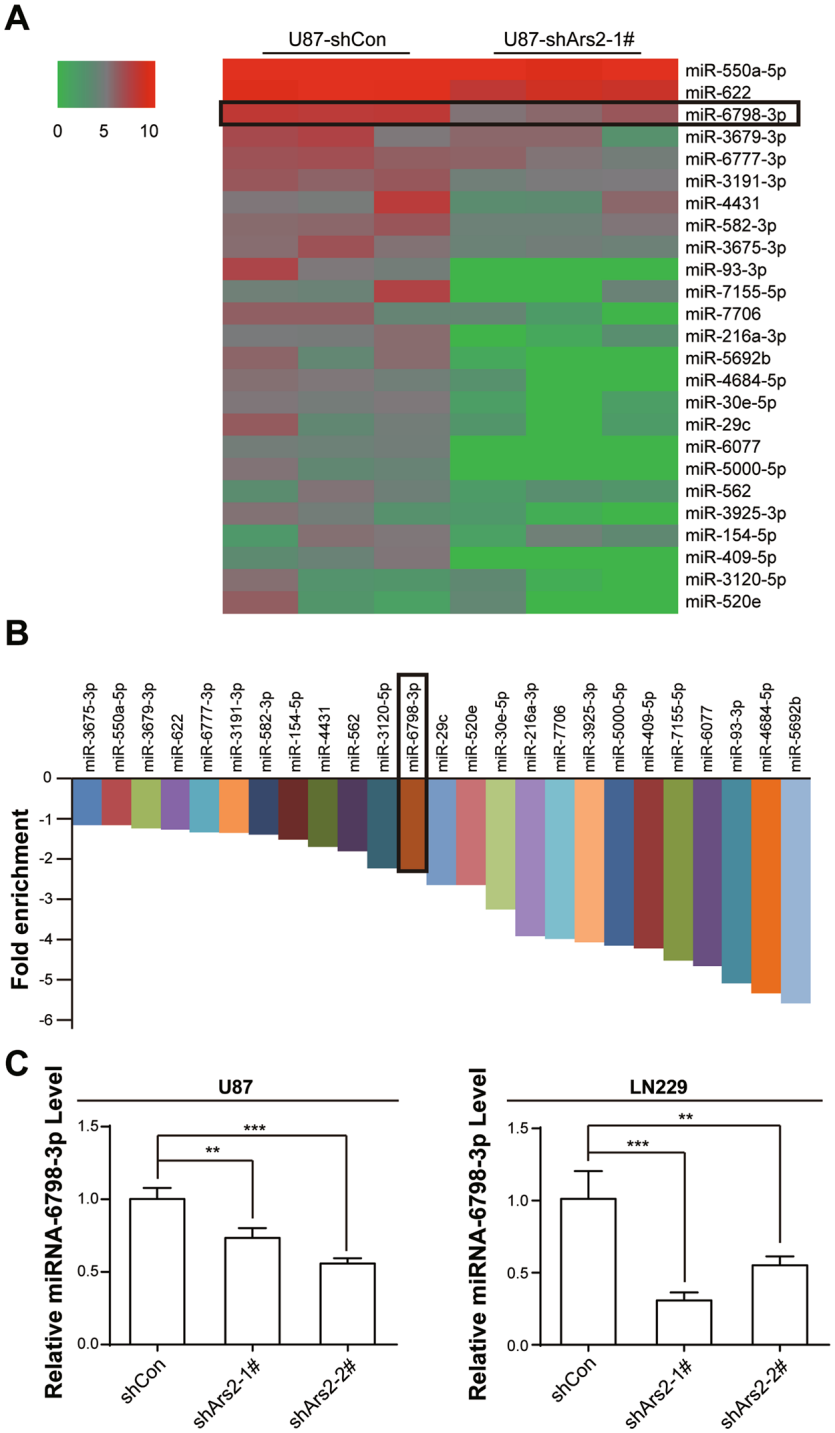
Depletion of Ars2 led to down-regulation of miR-6798-3p. (**A**) Heatmap showing differential miRNA expression between U87-shCon and U87-shArs2. (**B**) Analysis of down-regulated miRNAs in shArs2 cells and 25 down-regulated miRNAs (fold enrichment <−1) were shown. (**C**) Relative miR-6798-3p levels in U87 and LN229 cells transfected with either shCon or shArs2 (shArs2-1#, and shArs2-2#). Data were represented as the mean ± SD for three separate experiments, ^*^P < 0.05, ^**^P < 0.01, ^***^P < 0.001.

Since Ars2 is important for miRNA biogenesis and critical for cell proliferation^16–19,22,23^, we next analyzed the expression of miRNAs by performing high-throughput screening of all genomic miRNAs in U87 cells infected with vector control shRNA (shCon) or shArs2 using RiboArraymiDETECT MicroRNA Assay (Fig. 5A). After selection of those miRNAs whose expression was significantly down-regulated at least 2-fold (fold enrichment <−1), we selected 25 miRNAs as potential candidates in shArs2 cells compared to shCon cells (Fig. 5B). To further confirm down-regulation of miRNAs that are related to regulating cell proliferation and apoptosis after depletion of Ars2 using shRNA, a qRT-PCR analysis was employed. We found that the levels of miR-6798-3p was significantly down-regulated in U87 cells infected with Ars2 shRNA compared to that in vector control shRNA cells (Fig. 5C). Furthermore, overexpression of Ars2 markedly abrogated downregulation of miR-6798-3p mediated by Ars2 depletion (Fig. S2E). These events were also confirmed in another glioblastoma LN229 cells infected with Ars2 shRNA (Fig. 5C).

**Figure 6:**
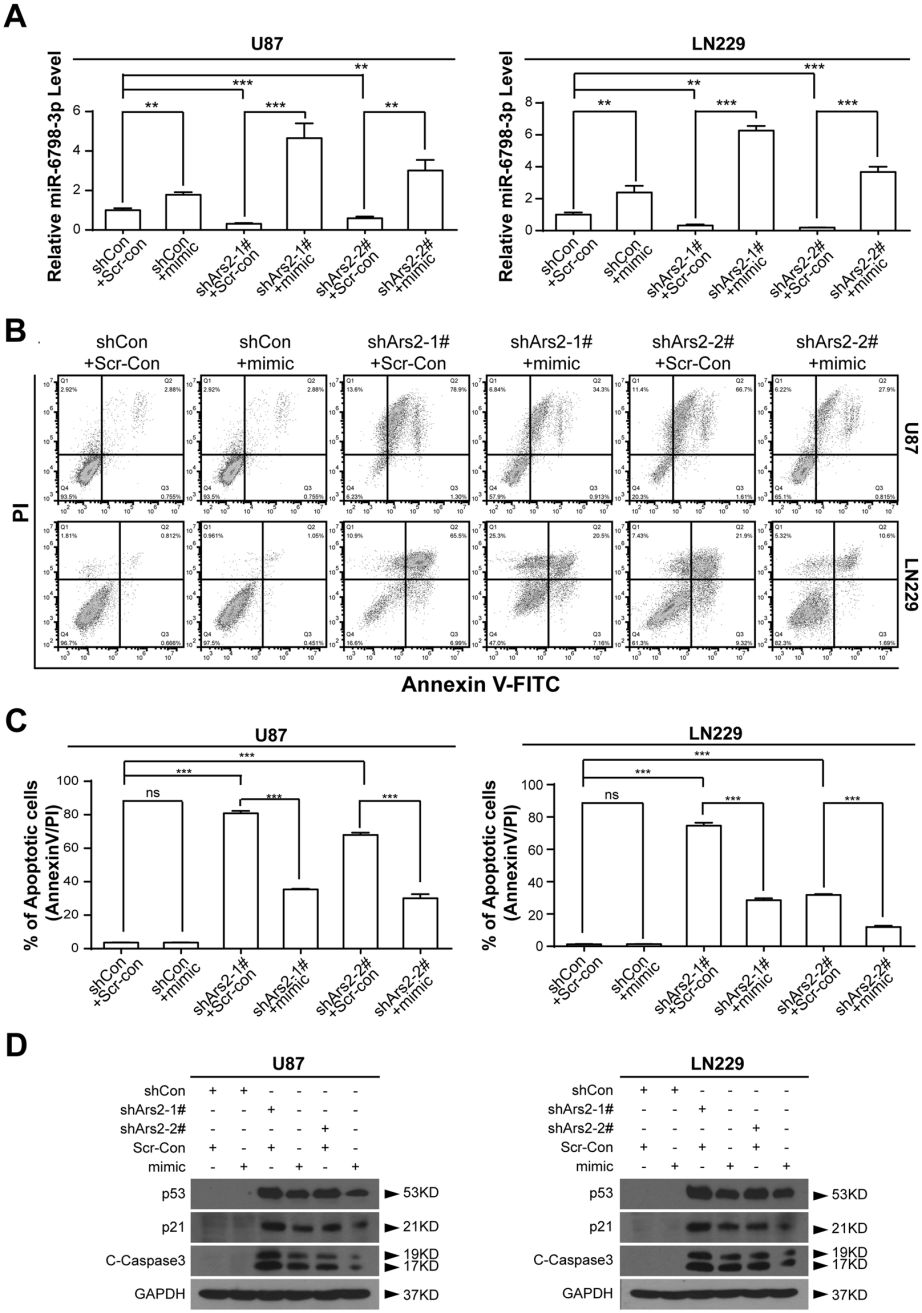
Cotransfection with miR-6798-3p mimic abrogated Ars2 depletion-mediated apoptosis in glioblastoma cells. shCon and shArs2 cells were cotransfected with miRNA-6798-3p mimic (50 nM) and scrambled control (Scr-Con, 50 nM). (**A**) Relative miRNA-6798-3p levels were determined by qRT-PCR analysis. (**B**,**C**) Cells were stained with Annexin V/PI, and the percentage of apoptotic cells was determined using flow cytometry. The values obtained from annexin V assays represent the means ± SD for three separate experiments. Statistical analysis was evaluated by Student’s t-test for three separate experiments, ^***^P < 0.001. (**D**) Western blot analysis was performed to determine the expression of apoptosis regulatory proteins including p53, p21, and C-Caspase 3. GAPDH levels were shown as loading control.

To further confirm that Ars2 depletion-mediated down-regulation of miR-6798-3p is involved in the induction of apoptosis in U87 and LN229 glioblastoma cells, miR-6798-3p mimic was employed. As shown in Fig. 6A, cotransfection with miR-6798-3p mimic significantly increased the expression of miR-6798-3p in either shCon or shArs2 cells. Furthermore, cotransfection with miR-6798-3p mimic markedly abrogated Ars2 depletion-mediated apoptosis (Fig. 6B). Western blot showed that cotransfection with miR-6798-3p mimic attenuated Ars2 depletion-mediated activation of caspase-3 and up-regulation of p53 and p21 (Fig. 6C). Taken together, these findings indicated that Ars2 depletion–mediated down-regulation of miR-6798-3p is involved in the induction of apoptosis through up-regulation of p53/p21.

### Ars2 Depletion Suppresses Tumor Growth in Orthotopic Glioblastoma Xenograft Model

**Figure 7:**
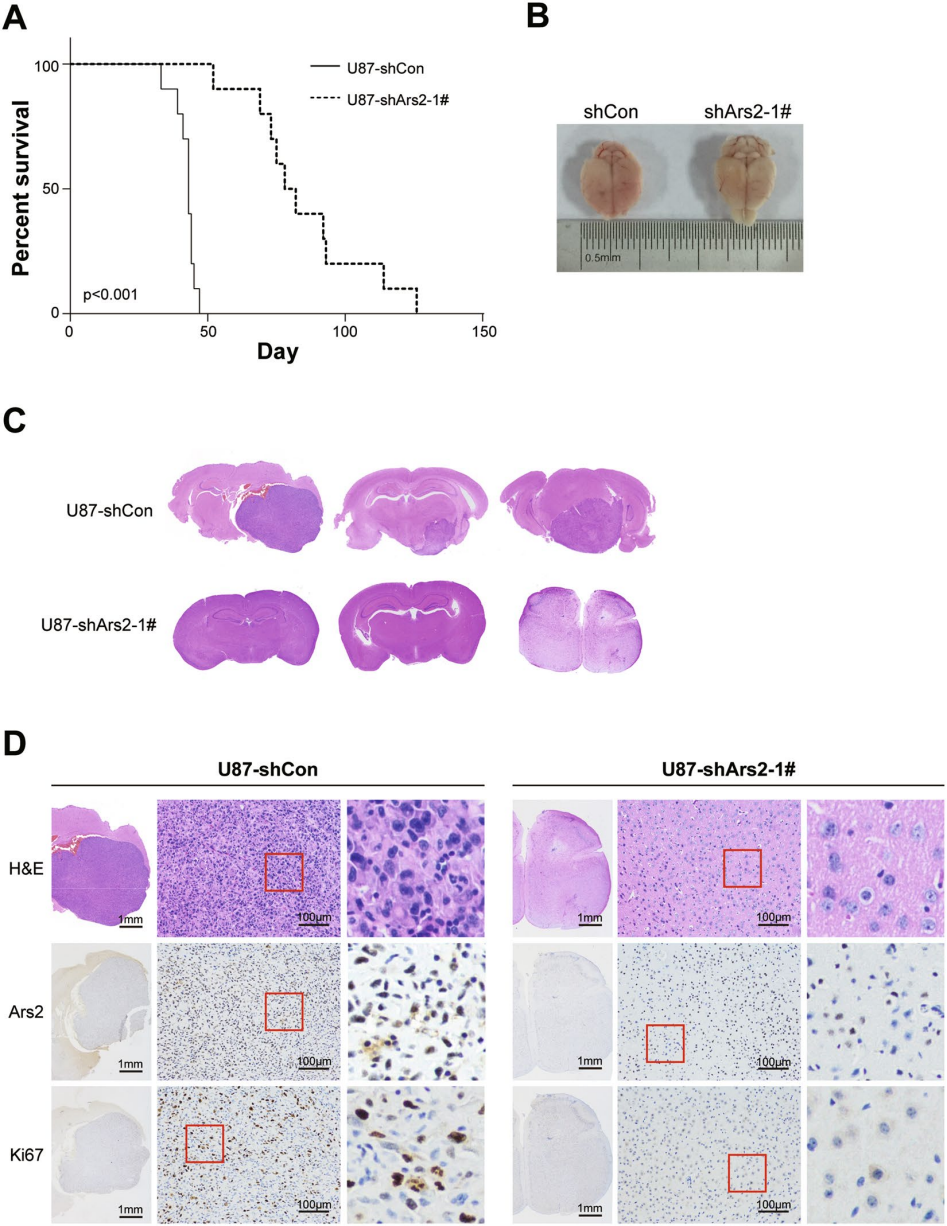
Depletion of Ars2 inhibited tumor growth in orthotopic glioblastoma xenograft model. (**A**) The survival time of U87-shArs2 glioma-bearing mice is significantly improved vs U87-shCon glioma-bearing mice. n = 10, ^***^P < 0.001 by the log-rank test for significance. (**B**) General observation showed that the brain swelling was presented in the mice of shCon group after 30 days of intracranial incubation. (**C**) Histological examination confirmed much slower tumor development in shArs2 glioma-bearing mice compared to shCon glioma-bearing mice. (**D**) H.E. staining, immunohistochemistry and Ki67 assay were performed to evaluate histological morphology, expression of Ars2, and cell proliferation in brain tissues of U87-shCon and U87-shArs2 glioma-bearing mice.

To evaluate the role of Ars2 in tumor growth of glioblastoma *in vivo*, we transplanted U87 glioblastoma cells into the brains of NOD/SCID mice to establish a orthotopic glioblastoma xenograft model. Kaplan-Meier survival analysis revealed that depletion of Ars2 with shRNA significantly prolonged the survival time of the NOD/SCID mice bearing U87 glioblastoma cells compared with that of vector control shRNA (median survival of 42.2 ± 3.9 days for control shRNA versus 82.2 ± 16.5 days for Ars2 shRNA, *P* < 0.001) (Fig. 7A). We also observed the effects of Ars2 depletion on tumor growth of glioblastoma. We found that the tumor volumes in NOD/SCID mice bearing U87 glioblastoma cells infected with control shRNA displayed large tumors extending throughout the whole right hemisphere, whereas mice bearing Ars2 shRNA cells displayed barely visible tumors (Fig. 7B,C).

To evaluate the effect of Ars2 depletion on the morphological changes in tumor section of orthotopic glioblastoma xenografts, H&E staining was employed. Histological examination showed that H&E-stained brain sections from mice bearing shCon cells displayed large numbers of tumor cells which occupy more than one lobe of a hemisphere or bilateral (Fig. 7C,D, top panels). In contrast, the brain section from mice bearing shArs2 cells displayed fewer tumor cells (Fig. 7D, top panels). As Ki-67 is an indicator for the ability of tumor cell proliferation, we then detected the levels of Ki-67 in the tumor section of orthotopic glioblastoma xenografts by using Ki-67 staining. As shown in Fig. 7D (middle panels), the increased expressions of Ki-67 were observed in brain sections from mice bearing shCon cells, whereas the expressions of Ki-67 were not observed in brain sections from mice bearing shArs2 cells. To determine whether Ars2 depletion was involved in inhibition of tumor growth, immunohistochemistry analysis was employed. The expression of Ars2 was markedly decreased in brain section from mice bearing shArs2 cells compared with that bearing shCon cells (Fig. 7D, bottom panels). Taken together, these findings indicate that Ars2 depletion was involved in the inhibition of tumor growth via inhibiting cell proliferation.

## Discussion

Arsenic resistance protein 2 (Ars2) was firstly found to be one of proteins could conferring resistance to arsenite on a Chinese hamster ovary cell line^16^, but the protein has remained poorly characterized. Recently, the overexpression of Ars2 was observed in human cholangiocarcinoma and hepato cellular carcinoma (HCC), suggesting that Ars2 could be identified as a prognostic and diagnostic indicator, even a potential target for therapeutic intervention^25,26^. Up to the present, the clinical significance of the Ars2 expression in human glioblastoma has not been explored. In this study, we reported for the first time that high expression of Ars2 predicted a decreased survival rate in glioblastoma patients. We also confirmed that high expressions of Ars2 at mRNA and protein levels were observed in multiple glioblastoma cell lines. It has been shown that Ars2 is essential for early mammalian development and plays a critical role in the proliferation of mammalian cells^29^. Consistent with previous reports, our studies revealed that overexpression of Ars2 promoted cell proliferation and colony formation in U87 and LN229 glioblastoma cells. In contrast, depletion of Ars2 with shRNA suppressed cell proliferation and colony formation *in vitro* and inhibited tumorigenicity *in vivo*. The functional analysis of Ars2 in cell proliferation showed that depletion of Ars2 caused cell cycle arrest at S phase, leading to inhibition of C2C12 myoblast progenitor cell proliferation^24^. However, our results indicated that depletion of Ars2 suppressed cell proliferation in glioblastoma cells through different mechanism. Depletion of Ars2 in glioblastoma cells with shRNA suppressed cell proliferation through induction of apoptosis but not cell cycle arrest. These events were also confirmed by the results of flow cytometry and western blot analysis, which indicated that depletion of Ars2 caused activation of caspase-3 and up-regulation of p53 and p21. Therefore, we speculated that Ars2 might be involved in glioblastoma progression and could be critical therapeutic target for glioblastoma intervention in the future.

Several lines of evidence support a role for Ars2 in RNA metabolism^16–24,30^. It has been shown that Ars2 contributes to some microRNAs (miRNA) biogenesis under cell proliferation signaling. Knockdown of Ars2 expression represses the biogenesis of several miRNAs that are important in cancer, let-7, miR-21, and miR-155 included^16,30^. However, the detailed mechanism of Ars2-regulated miRNA biogenesis in glioblastoma has not been fully elucidated. On the basis of microarray screening, we discovered that Ars2 depletion resulted in an extensive modification of the miRNA expression pattern in glioblastoma cells. This analysis defined a set of 25 miRNAs whose levels were decreased ≥ 2-fold by siRNA targeted to Ars2. Unlike previous findings indicated our study revealed that Ars2 depletion reduced the levels of miR-6798-3p, and Ars2 overexpression increased the levels of miR-6798-3p (Fig. S4), which is implicated in the regulation of proliferation and apoptosis in glioblastoma cells. In contrast, miR-21, let-7, and miR-155, these are implicated in the regulation of cellular proliferation, were not affected by depletion of Ars2 in glioblastoma cells. These data suggest that Ars2 may specifically regulate biogenesis of miRNA-6798-3p that is associated with cellular proliferation and apoptosis in glioblastoma cells.

Several lines of evidence support a role for Ars2 in the biogenesis of miRNAs that participate in the regulation of cellular proliferation. However, how Ars2-mediated miRNA biogenesis regulates cellular proliferation is not understood. It has recently been shown that the human genome may encode over 1000 miRNAs, which may target approximately 60% of mammalian genes in many human cell types^31–33^. He *et al*. reported that several tumor suppressors like phosphatase and tensin homolog deleted on chromosome ten (PTEN) and programmed cell death 4 (PDCD4) are directly regulated targets of miR-21^25^. The knockdown of Ars2 in cholangiocarcinoma cells decreased the level of miR-21, inhibited cell proliferation *in vitro* and tumor formation in nude mice *in vivo*. Ars2 depletion also led to an increase in PTEN and PDCD4 protein levels. These results suggest that Ars2 depletion increases PTEN and PDCD4 protein levels via the reduction of miR-21. Another tumor suppressor p53 was reported to be related with the biological activity of miRNAs, because p53 is, in itself, directly targeted by miRNA. More recently, it was shown that miR-34a is involved in the regulation of p53 activity and its downstream target p21 expression, leading to apoptosis^34^. The following results from this study strongly suggest that Ars2 depletion increases p53 activity and p21 expression via the reduction of miRNA-6798-3p, leading to inhibition of cellular proliferation and induction of apoptosis *in vitro*, as well as inhibition of tumor growth *in vivo*. (i) Ars2 depletion in glioblastoma cells (U87 and LN229) markedly decreased the expression of miRNA-6798-3p and increased the expression of p53 and p21, leading to inhibition of cellular proliferation and induction of apoptosis; (ii) Cotransfection of Ars2-knockingdown cells with miRNA-6798-3p mimic attenuated Ars2 depletion-inhibition of cellular proliferation and induction of apoptosis through increases in levels of miRNA-6798-3p and decreases in the expression of p53 and p21. Taken together, these findings suggest that Ars2-mediated miRNA-6798-3p progression is required for cellular proliferation and tumorigenicity in glioblastoma.

In conclusion, the present study provided the important evidence that Ars2 has an important role in the regulation of cellular proliferation and tumorigenesis in glioblastoma. Ars2 depletion reduced the levels of miRNA-6798-3p, leading to up-regulation of p53 and p21, and culminating in inhibition of cellular proliferation and induction of apoptosis *in vitro* and inhibition of tumorigenicity *in vivo*. These findings suggest that Ars2 may act as a critical regulator of cell proliferation and tumorigenicity, and seems to be a novel and promising target for the treatment of glioblastoma.

## Materials and Methods

### Cell Culture

The human glioblastoma cell lines (LN229, U87, A172, U118, and U251) were purchased from the American Type Culture Collection (ATCC, Manassas, USA). All of these cells were cultured in DMEM/F12 medium (Invitrogen, Waltham, USA) supplemented with 2 nM L-Glutamine, 10% fetal bovine serum (Gibco, Waltham, USA), 100 U/mL penicillin and 0.1 mg/mL streptomycin. 293FT cell line was purchased from Thermo Fisher Scientific (New York, USA), was cultured in DMEM(Invitrogen, Waltham, USA) supplemented with 10% fetal bovine serum, 1% P/S, 0.1 nM Non-Essential Amino Acids, 0.5 mg/mL G418, 4mL L-Glutamine, and 1 mM MEM sodium pyruvate. Human astrocytes (HA) and growth medium were from ScienCell Research Laboratories (Carlsbad, CA). All of these cells were maintained in 5% CO_2_ at 37 °C.

### Antibodies

Mouse monoclonal anti-Ars2 and rabbit polyclonal anti-p53 antibodies were purchased from Santa Cruz Biotechnology (Dallas, USA). Purified mouse anti-p21antibody was purchased from BD Pharmingen (Franklin Lakes, USA). Rabbit Ab anti-Cleaved Caspased-3 was purchased from Cell Signaling Technology (Boston, USA). Mouse monoclonal anti-Glyceraldehyde-3-phosphate dehydrogenase (GAPDH) antibody was purchased from Beyotime Technology (Shanghai, China). Anti-Ki67, C-Terminal antibody produced in rabbit, was purchased from Sigma Aldrich (St. Louis, USA). Primary antibodies were used at a dilution of 1:1000 for Western Blotting. Primary antibodies were used at a dilution of 1:500 for IHC.

### RNA Extraction and Quantitative Real-time Polymerase Chain Reaction

Total RNA was isolated using TRIzolreagent (Invitrogen, Waltham, USA), according to the manufacturer’s instructions. qRT-PCR was performed in triplicate in IQ2 PCR System (Bio-Rad Laboratories, Hercules, California, USA). miRNA levels were measured using the Bulge-Loop^TM^miRNAqRT-PCR Primer and Starter Kit (RIBOBIO, Guangzhou, China), and mRNA levels of Ars2 were detected using in accordance with the TaKaRa manufacturer’s instruction. Ars2 forward primer sequences (GGTGACCTTCGACCGCAGTGTT), Ars2 reverse primer sequences (TGGGTGATGCCGTTGATGTTGC), β-Actin forward primer (TGACGTGGACATCCGCAAAG), and β-actin reverse primer sequences (CTGGAAGGTGGACAGCGAGG) were purchased from Huada Gene Research Institute (Beijing, China). 2-deltadeltaCt-method was used to analyse the real-time PCR data. U6 and β-actin was used for normalization of the microRNA and mRNA expression, respectively.

### Oligonucleotide, Ars2 Overexpression, and Knockdown Plasmid Synthesis and Transfection

Has-miR-6798-3p mimic (CUACCCCCAUCCCCCUGUAG) and microRNA-scrambled control were purchased from RIBOBIO. shCon, shArs2-1#, and shArs2-2# sequences were inserted into the pLKO.1 vector. Human full-length Ars2 cDNA fragment was subsequently cloned into pCDH-CMV-MCS-EF1-puro vector to construct the overexpression plasmid. miRNA sequences were transfected into cultured cells using Lipofectamine 3000 regant (Invitrogen) according to the manufacturer’s instructions. The shArs2-1#, shArs2-2#, shCon, Ars2 overexpression and vector control (Con) plasmids were transfected into 293FT cells using the Lipofectamine 3000 reagent (Invitrogen), the Lentivirus was infected into glioblastoma cells. After that, the transfected cells were selected with 2–4 μg/mL puromycin for 1 week. p53, p21, and control siRNA (sip53, sip21, and siCon) were purchased from Santa Cruz Biotechnology. siRNA were transfected into U87 and LN229 cells using the Lipofectamine 3000 regent according to the manufacturer’s instructions.

### Softagar Assay

1000 cells were mixed with 0.3% Noble agar in cell culture medium and plated these cells in 24-well plates which contained a solidified bottom layer (0.6% Noble agar in growth medium). And then, the colonies were photographed after 21 days. After treatment, MTT were added to each well and maintained in 37 °C for 30 min till the white clone turned black, then the number of colonies were counted and statistically analyzed.

### Cell Apoptosis Assay

Glioblastoma cells were stained with Annexin V-FITC and PI to evaluate by flow cytometry on the basis of the manufacturer’s protocol (BD Biosciences PharMingen). Briefly, glioblastoma cells were collected and washed with ice-cold PBS for twice, and then stained with annexin V-FITC (5 μL) and 10 μL of PI (50 μg/mL) in binding buffer (pH 7.4, 10 mM HEPES, 140 mMNaOH, 2.5 mM CaCl_2_) for a quarter at room temperature in the dark. Quantification analysis of apoptotic U87 and LN229 cells was performed by flow cytometry using a FACScancytofluorometer (BD Biosciences).

### miRNA Assay

U87-shCon and U87-shArs2-1# cells were seeded in 10 cm dishes and cultured. Total RNA was extracted from U87-shCon and U87-shArs2-1# cells using TRIzol reagent (Invitrogen) in accordance with the manufacturer’s protocal. Then, the expressions of miRNA levels were detected with RiboArraymiDETECT MicroRNA Assay (RiboBio).

### Cell Viability Assay

To test cell viability, 1000 cells/well were cultured in 96-well plates 5% CO_2_ at 37 °C for 5 days, and MTT assay was performed in accordance with the manufacturer’s instruction. All experiments were performed independently at least in triplicate.

### Western Blot Assay

Proteins were extracted from glioma cells, and cells were lysed in RIPA buffer (Beyotime, China, P0013). Equal amounts of each sample were separated by SDS-PAGE gels and transferred to a ployvinylidenedifluoride membrane (Bio-Rad, 162-0177). Blocked the blots with 5% nonfat dried milk, and then the blots were incubated with primary antibodies overnight at 4 °C. The bands were washed by TBST for three times, and incubated with horseradish peroxidase-conjugated antibodies (Kirkegaard and Perry Lboratories, Gaithersoburg, MD, USA). At last, these bands were visualized with Clarity Western ECL Substrate (Bio-Rad, 170-5061).

### *In Vivo* Tumorigenic Assay

Orthotopic implantations were employed to the *in vivo* study. Twenty-six NOD/SCID mice (35-day old) were intractranially injected into the brains with 1 × 10^5^ cells (2 mm lateral and 1 mm anterior to the bregma, 3.5 mm deep) and placed into a stereotaxic frame. The survivorship curve was draw until the last mouse had died. After injected 30 days, the original tumors were photographed, and brains were collected, fixed in neutral buffered 4% PFA solution, and embedded in paraffin. Immunohistochemical (IHC) analysis and Hematoxylin and eosin (H&E) staining were employed to histopathological evaluations of the tissues by Servicebio Technology (HONG KONG, China). Briefly, the tumor sections were incubated with primary antibodies for Ars2 (1:200) and for Ki-67 (1:500), followed by detection using the ChemMate Detection kit (Dako, Denmark).Positive reaction was indicated by brown color using DAB, and the cells were counterstained with hematoxylin.

All mice were monitored and raised under the specific pathogen-free (SPF) conditions. And the welfare and experimental procedures of mice were carried out according to the Guide for the Care and Use of Laboratory Animals (Ministry of Science and Technonlogy of China, 2006) and approved by the animal ethics committee, Southwest University.

### Statistical Analysis

Data were represented as means ± SD. For comparison between two data sets, a Student’s t test was used. For analysis of three or more sets of data, ANOVA was used. For analysis of survival rate, log-rank test was used. *P < 0.05, **P < 0.01, or ***P < 0.001 was considered statistically significant.

## Electronic supplementary material

Supplementary Dataset 1
