# Supplementary material for: Ars2 promotes cell proliferation and tumorigenicity in glioblastoma through regulating miR-6798-3p
Source: Sci Rep. 2018 Oct 22;8:15602. doi: 10.1038/s41598-018-33905-x (PMC6197286; doi:10.1038/s41598-018-33905-x)
Supplement: Supplementary file 1 — Supplementary Dataset 1 [file 41598_2018_33905_MOESM1_ESM.docx]

**Ars2 promotes cell proliferation and tumorigenicity in glioblastoma through regulating miR-6798-3p**

Yibiao Chen^†1^, Xiaoye Hu^†2^, Yunong Li^2^, Hongwei Zhang^2^, Ruoqiu Fu^2^, Yanxia Liu^2^, Jinjiao Hu^2^, Qin Deng^2^, Qingsong Luo^2^, Dunke Zhang^1^, Ning Gao^2^, Hongjuan Cui^1^

^1^State Key Laboratory of Silkworm Genome Biology, Southwest University, Chongqing, China (C.Y., Z.D., C.H.)

^2^College of Pharmacy, Third Military Medical University, Chongqing, China (H.X., L.Y., Z.H., F.R., L.Y. H.J., D.Q., L.Q., G.N.)

**Running title:** Role of Ars2 in glioblastoma proliferation

**Corresponding Authors:** Hongjuan Cui, Ph.D., State Key Laboratory of Silkworm Genome Biology, Southwest University, 2^#^Tiansheng Road, Beibei District, Chongqing 400716, China ([hongjuan.cui@gmail.com](mailto:hongjuan.cui@gmail.com)); Ning Gao, Ph.D., College of Pharmacy, Third Military Medical University, 30 Gaotanyan Street, Shapingba District, Chongqing 400038, China ([gaoning59@tmmu.edu.cn](mailto:gaoning59@tmmu.edu.cn) or gaoning59@163.com)

^†^These authors contributed equally to this work

**
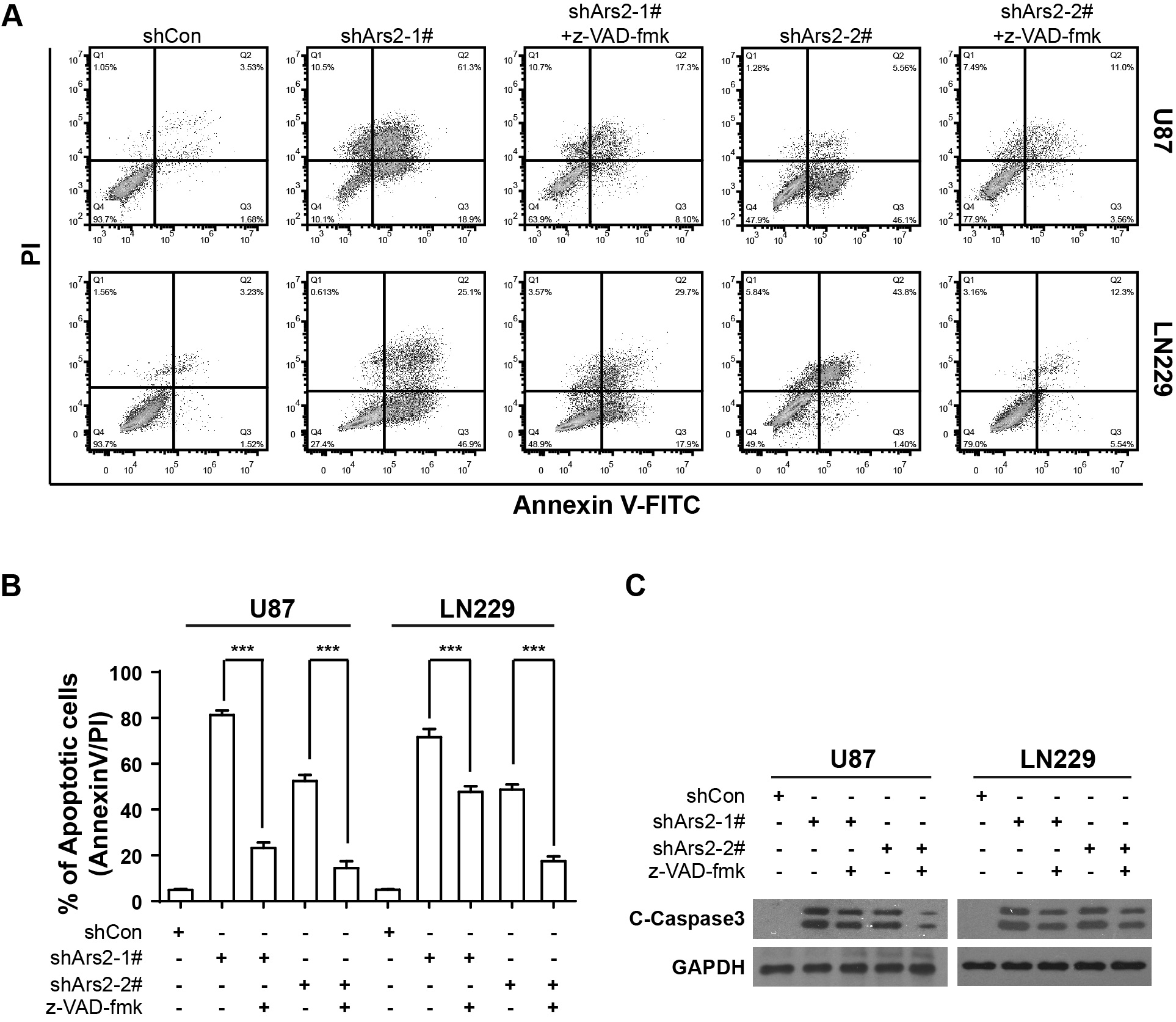
Fig.S1. Inhibition of caspase activation by z-VAD-fmk attenuated apoptosis induced by depletion of Ars2 in glioblastoma cells.** U87 and LN229 cells were transfected with vector control siRNA (shCon) and Ars2 siRNA (shArs2-1#, and shArs2-2#), after which cells were treated by an irreversible pan-caspase inhibitor z-VAD-fmk (25 μmol/L). (A) The percentage of apoptotic cells was determined by flow cytometry using Annexin V/PI staining. (B) Statistical analysis of the cellular apoptosis levels. Data were represented as the mean ± SD for three separate experiments, ****P* < 0.001. (C) Total cellular extracts were prepared and subjected to Western blot analysis using antibodies against C-Caspase 3. GAPDH levels were shown as loading control.

**
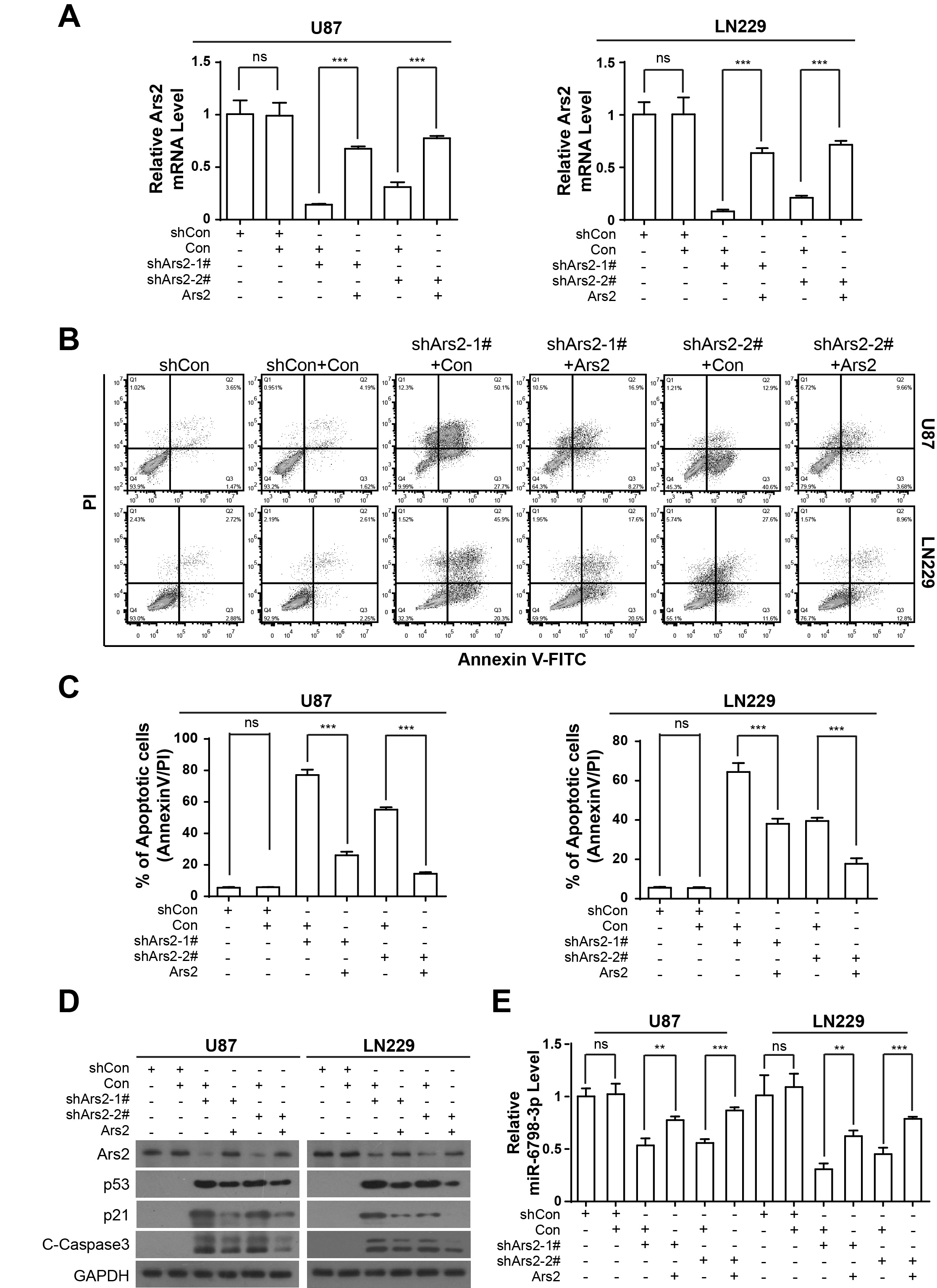
Fig.S2. Overexpression of Ars2 abrogated Ars2 deletion-mediated apoptosis in glioblastoma cells.** shCon and shArs2 cells were cotransfected with Ars2 and vector control (Con). (A) Relative Ars2 mRNA expression was determined by qRT-PCR analysis. (B and C) Cells were stained with Annexin V/PI, and the percentage of apoptotic cells was determined by using flow cytometry. (D) Total cellular extarcts were prepared and subjected to Western blot analysis using antibodies against Ars2, p53, p21, and C-Caspase 3. GAPDH levels were shown as loading control. (E) Relative miRNA-6798-3p levels were determined by qRT-PCR analysis. All data were represented as the mean ± SD for three separate experiments, ***P* < 0.01; ****P* < 0.001.


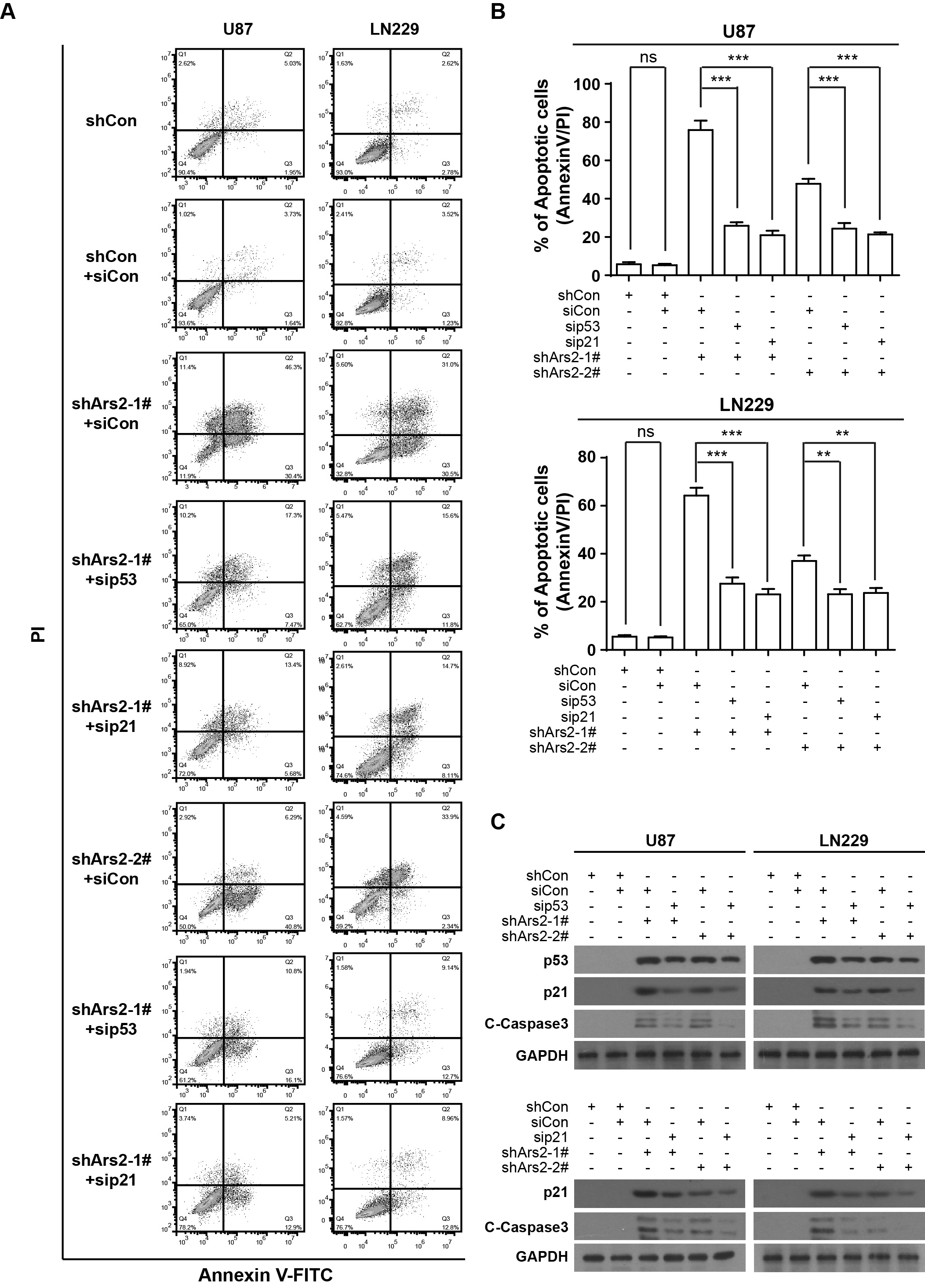


**Fig.S3. Knockdown of p53 and p21 with siRNA abrogated Ars2 deletion-mediated apoptosis in glioblastoma cells.** shCon and shArs2 cells were cotransfected without or with p53 and p21 siRNA (si-p53 and si-p21). (A and B) Cells were stained with Annexin V/PI, and the percentage of apoptotic cells was determined by using flow cytometry. All data were represented as the mean ± SD for three separate experiments, ***P* < 0.01; ****P* < 0.001. (C) Total cellular extracts were prepared and subjected to Western blot analysis using antibodies against p53, p21, and C-Caspase 3. GAPDH levels were shown as loading control.

**
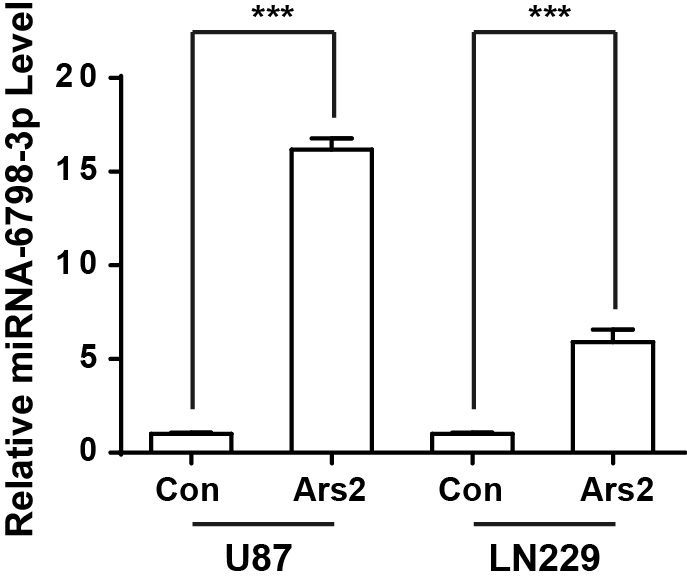
**

**Fig.S4. Overexpression of Ars2 increased the levels of miR-6798-3p.** U87 and LN229 cells were transfected with vector control or Ars2, after which the levels of miR-6798-3p were determined by qRT-PCR analysis. All data were represented as the mean ± SD for three separate experiments, ****P* < 0.001.
